# Development and characterization of novel flexible cellulose electrodes for electrophysiological monitoring

**DOI:** 10.1039/d5ra03473f

**Published:** 2025-07-18

**Authors:** Meera Alex, Amani Al-Othman, Mohammad Al-Sayah, Hasan Al-Nashash

**Affiliations:** a Biosciences and Bioengineering Graduate Program, American University of Sharjah Sharjah United Arab Emirates; b Department of Chemical and Biological Engineering, American University of Sharjah Sharjah United Arab Emirates aalothman@aus.edu; c Department of Biology, Chemical and Environmental Sciences, American University of Sharjah Sharjah United Arab Emirates; d Department of Electrical Engineering, American University of Sharjah Sharjah United Arab Emirates

## Abstract

Flexible and biocompatible electrodes are crucial components in developing future wearable and implantable biomedical devices. In this work, a novel composite bioelectrode from cellulose, supported by polydimethylsiloxane (PDMS), and glycerol were developed. Cellulose, an abundant and biodegradable biopolymer, provides the conductivity, while PDMS provides the support and the mechanical elasticity. All together, they provide elasticity and skin-conformability. Glycerol was added in this work as it also acts as an ionic conductor and a plasticizer, thus, improving interfacial charge transfer and electrode hydration stability. The composite was fabricated by an easy polymerization and curing process. The morphological, electrochemical, and mechanical characteristics of the fabricated electrode were evaluated. Electrochemical impedance spectroscopy (EIS) and cyclic voltammetry (CV) showed low impedance, high conductivity, and stability. The electrochemical characteristics demonstrated the lowest bulk resistance of 0.658 kΩ, a conductivity of 0.0193 S m^−1^ and a charge storage capacity of 4.626 mC m^−2^. The ductile properties for the samples showed a low elastic Young modulus of 10.3 ± 5.4 kPa. Electroencephalograph (ECG) signal was recorded at a considerable good quality with SNR of 33.31 dB. Thus, the cellulose–PDMS–glycerol electrode material appears to offer a highly promising, green platform for the development of soft bioelectronics to be employed in real-time physiological signal monitoring.

## Abstract 2

Flexible and biocompatible electrodes are crucial components in developing future wearable and implantable biomedical devices.

## Graphical abstract

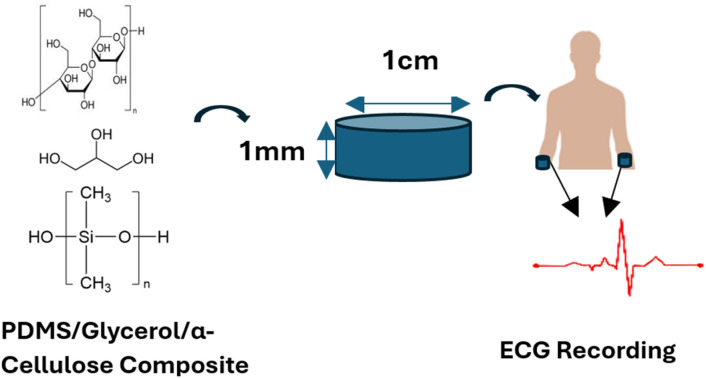

## 1. Introduction

Bioelectrodes act as transducers, facilitating the interface between injured nerves and active muscles. Such bioelectrodes are widely used for flexible and implantable applications. In recent years, flexible electronics comprising of epidermal, wearable and implantable electrodes are widely used for many applications such as brain computer interface, human–machine interactions, and continuous health monitoring.^1,2^ Biopotential signals recorded from skin surface, including electromyography (EMG), electrocardiography (ECG), and electroencephalography (EEG) play vital roles in assessing human function and medical diagnosis.^3^ Therefore, accurate monitoring of these signals is crucial for integrating wearable devices into everyday healthcare.^3^ Here comes the role of flexible electrodes that serves as the interface to capture electrical signals from the body at the skin interface or interfacing directly with neurons or muscles. To date, metals and conductive polymers have dominated bioelectrode fabrication for flexible and implantable applications. The commercial metal-based wet electrodes (Ag/AgCl) have dominated the field of body surface electrophysiological monitoring because of their affordability and strong adhesion.^3^ However, they also suffer from challenges such as the need for skin preparation, lack of flexibility, potential user discomfort, and gel drying over time.^3^ Gold and platinum are also amongst the metal electrodes used in implantable applications. When it comes to implantation, they also suffer from mechanical mismatch with soft tissues like nerves and increase in resistance with time. Besides, the chemical and mechanical properties of these materials differ significantly from those of biological tissues.^4^ The next widely researched flexible bioelectrodes are based on conductive polymers such as polyaniline (PANI), poly(3,4-ethylenedioxythiophene) PEDOT, polyimide (PI) *etc.* Although conductive polymers offer high conductivity and mechanical strength, they are often costly and non-biodegradable. This advocates the need for the development of a biocompatible, low cost, flexible and conductive electrode. One such widely used flexible component is polydimethylsiloxane (PDMS), which exhibits compatibility with biological tissues but lacks the requisite electrical conductivity. Most researchers have exploited the flexible nature of PDMS and incorporated other materials such as carbon dots^5^ and Mxene^6^*etc.* to achieve conductivity and flexibility at the same time.

In recent years, natural biopolymers like chitosan, collagen, silk, gelatin, and cellulose have attracted considerable interest for their green nature, and potential use in flexible devices, such as sensors and portable energy storage systems.^4^ Among this, cellulose has gained much attention in many research studies. Cellulose possesses structural and functional characteristics that make it a highly suitable material for sensor applications.^7^ Cellulose is a widely available organic compound that serves as a key structural element in many green plants. The glucose units in this polymer include six free hydroxyl groups that participate in both intra- and inter-chain hydrogen bonding.^8^ The extensive hydrogen bonding network stabilizes the structure and facilitates proton transfer.^9^ This hydrogen bonding network also enables ionic conductivity in the cellulose-based materials.^10^ Cellulose and its derivatives are optimal substrates for sensing devices due to several key features, including a high density of surface hydroxyl groups, large specific surface area, high aspect ratio, significant crystallinity, excellent mechanical strength, and superior thermal stability.^8,11^ They also possess piezoelectric and dielectric properties.^12^ This has been successfully demonstrated by Chandrashekar *et al.*,^13^ with an innovative cellulose/PDMS biomechanical energy harvesting device designed for self-powered and wearable electronic applications. Various forms of cellulose are used for multifunctional wearable sensor applications.^8^ They often incorporate components like conjugated polymers, metals, and semiconductors into the cellulosic substrate for better performance. But this compromises biocompatibility of flexible/wearable device particularly for health monitoring.^8^

Although there is a growing interest in the application of sustainable and biocompatible materials for the development of bioelectrodes, cellulose based electrodes are not well studied regarding their attractive electrochemical properties, flexibility, and long-term stability when used under physiological conditions. Most studies are focused on mechanical properties of cellulose composites or surface treatment. While ionic liquids such as [Amim]Cl, [Bmim]Cl, and [Emim][Ac]^14^ have been employed to enhance ionic conductivity, their potential cytotoxicity impedes their suitability for implantable applications. Additionally, complete knowledge of the influence of the structural modifications by addition of plasticizers like glycerol on the electrochemical characteristics, signal quality acquisition, and bio interface compatibility of cellulose-based electrodes is limited. This work aims to fill this gap by designing and characterizing a new composite electrode based on cellulose with glycerol embedded in PDMS. In here we explore the potential of cellulose as a promising and sustainable material for bioelectrode synthesis and for electrophysiological monitoring applications. Thus, we propose a simple one-pot synthesis method in which cellulose acts as an additive to PDMS along with glycerol. Glycerol acts as a plasticizer for improving conductivity of the prepared nanocomposites. Reports from recent research study by Abdullah *et al.*^15^ confirms that 40% addition of glycerol improves the DC conductivity in sodium-conducting polyelectrolyte. Addition of cellulose provides a large surface area of hydroxyl groups, leading to the formation of extensive hydrogen bonding networks.^16^ It offers exceptional characteristics, such as biocompatibility, biodegradability, and environmental friendliness. The properties of cellulose, including a Young's modulus akin to neural and skin tissues, chemical modifiability for enhanced durability and conductivity, make it an attractive candidate for bioelectrode fabrication. Ultimately the main goal of this study is to test the performance of cellulose/PDMS-based bioelectrode as a sustainable alternative to metal-based counterparts.

## 2. Materials and methods

### 2.1 Materials

The materials used for the fabrication of the bioelectrode included α-cellulose powder (P code: 102443378, CAS no. 9004-34-6) from Sigma-Aldrich, polydimethylsiloxane (PDMS) prepared using Sylcap™ 284-S (standard cure) silicone elastomer base and curing agent from Microlubrol, and glycerol (CAS# 56-81-5) from Thermo-Fisher Scientific.

### 2.2 Fabrication of bioelectrode

**Fig. 1:**
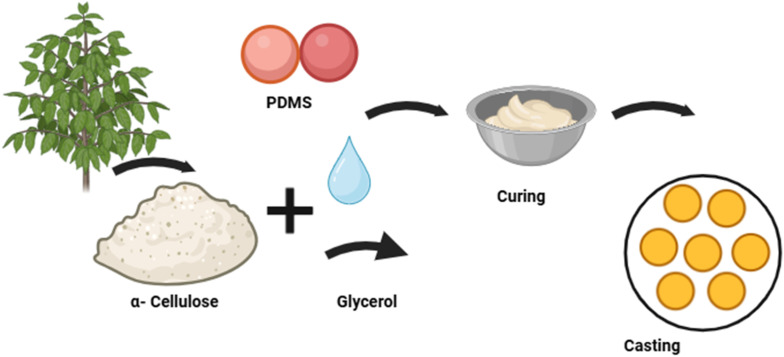
Fabrication process of α-cellulose/PDMS electrode.

**Fig. 2:**
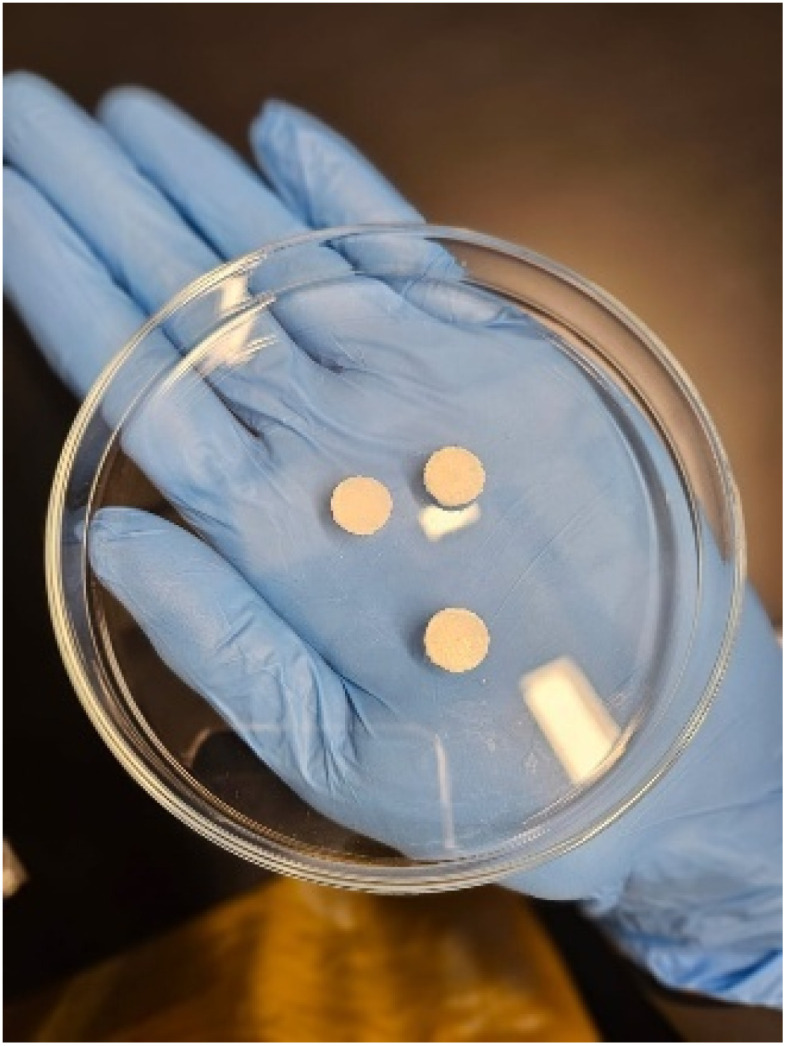
A picture shows the dimensions of the obtained/fabricated electrode samples for sensing electrophysiological signal.

Fabrication process of cellulose-based electrode included mixing of PDMS elastomer with the curing agent in a 9 : 1 ratio at room temperature. α-Cellulose powder and glycerol were then added and thoroughly mixed to achieve a homogeneous blend. To eliminate air bubbles, the mixture underwent desiccation. Further to desiccation, the PDMS/α-cellulose/glycerol mixture was casted into a custom-designed tray with specific dimensions for curing as depicted in the Fig. 1, and the samples are shown in Fig. 2. A single electrode dimensions include 1 mm thickness and cross-sectional area of 0.785 cm^2^.

### 2.3 Morphological, electrochemical, and mechanical characterization

To assess the surface morphology and structural features of the fabricated materials, scanning electron microscopy (SEM) was conducted using a TESCAN MAGNA system (TESCAN, Czech Republic). This analysis enables the observation of surface roughness, porosity, and microstructural organization of the sample. For the chemical characterization, Fourier Transform Infrared (FT-IR) spectroscopy was employed to detect the functional groups present in the sample. FTIR was performed using a Shimadzu IRTracer-100 spectrometer, which provides insight to chemical bonding within the material.

Electrochemical impedance spectroscopy (EIS) is a widely used technique for analysing how a material responds to an applied electric field, either at a fixed or varying frequency. It is particularly useful for characterizing the electrical properties of coatings and soft materials. In this method, an alternating current (AC) signal is applied to the sample, and the resulting voltage and current responses are recorded to calculate both the real and imaginary components of impedance. These impedance values are then plotted over a range of frequencies to analyse the material's behaviour. In this study, EIS measurements were carried out using a Biologic SP-200 potentiostat (Seyssinet-Pariset, France). The experimental setup included a custom-designed cell consisting of two stainless steel electrodes, each with a cross-sectional area of 0.7854 cm^2^. The sample was securely positioned between the electrodes during testing. A sinusoidal voltage of 10 mV was applied across a frequency range from 100 mHz to 7 MHz data acquisition and analysis were performed using EC Lab software (version 11.02).

Electrochemical characterization involved two tests aimed at evaluating the electrical conductivity and charge storage capacity (CSC) of the materials. The conductivity was determined by measuring the bulk impedance through electrical impedance spectroscopy (EIS) and as obtained from the EC-lab software. It can be also done by extrapolating at the high frequency region to find the intercept with the *x*-axis. SP-200 Biologic Potentiostat in conjunction with EC-Lab software (version 11.02) was used for electrochemical characterization. During the EIS test, the sample was sandwiched in a custom-made cell with two stainless-steel electrodes, and a small AC voltage of 10 mV was applied over a frequency range from 100 mHz to 7 MHz. This allowed the determination of impedance behaviour and charge transport properties. Additionally, cyclic voltammetry (CV) was performed using the same electrochemical workstation. A voltage sweep ranging from −1 V to +1 V was applied at a scan rate of 20 mV s^−1^ to assess the charge storage capability and electrochemical stability of the fabricated electrodes.

The mechanical properties of the samples were evaluated through a quasi-static uniaxial tensile test using the 5582 Universal Testing System (Instron, USA), following the ASTM D638 standard. The test was conducted on the most consistent triplicate set of samples. During the test, the specimens were subjected to a gradually increasing tensile force under controlled conditions until failure occurred. This study was performed with this analysis provided crucial data on the material's tensile strength, elasticity, and mechanical robustness.

## 3. Results and discussion

### 3.1 Surface structure and morphology

**Fig. 3:**
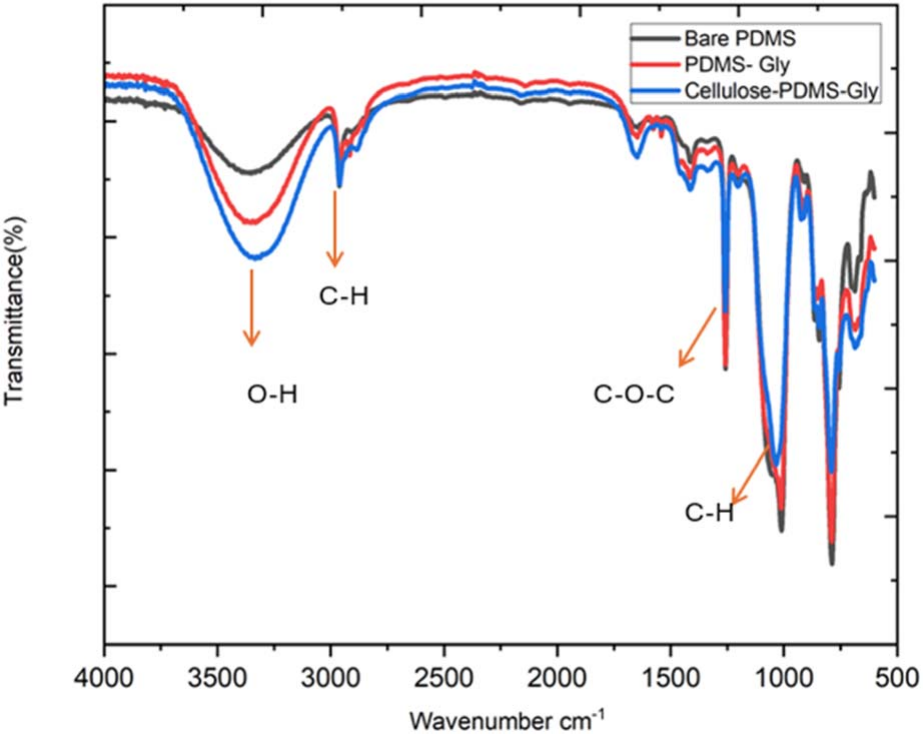
ATR-FTIR spectra of three different composite formulations (bare PDMS, PDMS-GLY and cellulose-PDMS-GLY), highlighting characteristic absorption bands corresponding to O–H stretching (∼3300 cm^−1^), C–H stretching (∼2900 cm^−1^), and C–O–C stretching (∼1050 cm^−1^).

To verify the successful incorporation of α-cellulose and glycerol within the nanocomposite electrode following fabrication, attenuated total reflection Fourier-transform infrared spectroscopy (ATR-FTIR) was conducted. Spectral analysis was performed across three sample types: bare PDMS, PDMS/glycerol, and the full α-cellulose/30% cellulose/64% PDMS/6% glycerol composite (best performing electrode sample-most conductive). This comparative approach allowed for the identification of characteristic absorption peaks corresponding to each component in the composite system as depicted in Fig. 3. The ATR-FTIR spectra clearly exhibited distinctive peaks attributable to PDMS, α-cellulose, and glycerol, confirming their presence within the fabricated films. Specifically, a broad absorption band observed in the range of 3000–3500 cm^−1^ was assigned to O–H stretching vibrations,^17^ indicating the presence of hydroxyl groups, primarily from glycerol and α-cellulose. Peaks in the region of 2800–3000 cm^−1^ corresponded to C–H stretching vibrations, which are typical of the PDMS backbone.^17^ A prominent absorption band around 1200 cm^−1^ was attributed to C–O–C stretching, while a band near 700 cm^−1^ corresponded to C–OH bending modes.^17^ These spectral features agree with previously reported data and support the presence of the targeted functional groups within the composite structure. Among the three samples, the α-cellulose/PDMS/glycerol composite exhibited a notably more intense O–H stretching band, indicating a higher concentration of hydroxyl groups. This observation suggests strong interactions among the components, particularly hydrogen bonding between α-cellulose and glycerol within the PDMS matrix, as illustrated in Fig. 3. Overall, the FTIR analysis confirms the presence of all intended constituents and validates the successful formation of the composite structure through characteristic functional group identification.

**Fig. 4:**
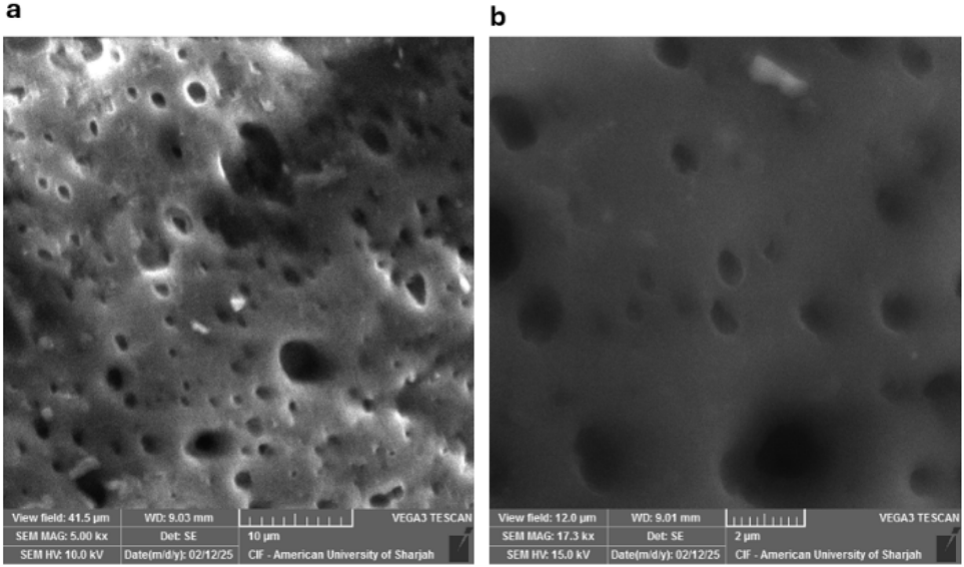
SEM images for (a) 10 μm resolution (b) 2 μm resolution for the 30% cellulose/64% PDMS/6% glycerol sample.

Fig. 4 shows a scanning electron microscope image of the best performing electrode composition 30% cellulose/64% PDMS/6% glycerol. The images in Fig. 4a and b are a top-view picture of the formed surface electrode at magnifications of 10 μm and 2 μm resolutions respectively for this electrode. Fig. 4a reveals the pores likely resulting from the interaction of glycerol and cellulose during the curing or mixing process. The high-resolution micrographs are illustrative of the nanocomposite material surface morphology and structure. The SEM examination reveals a relatively smooth yet porous surface characteristic of the PDMS matrix. In the matrix, the entrapped α-cellulose is visible, indicating that the cellulose fibres were incorporated well during processing. The cellulose appears to form a network of micro-scale crosslinks in the PDMS substrate, suggesting considerable physical interactions and possible interfacial bonding between the polymer and the natural filler. This morphology, which is achieved by entrapping the cellulose and incorporating glycerol as a plasticizer, is believed to enhance the overall electrical performance of the composite. The porous morphology can facilitate ionic mobility in nanocomposites.^18^ In here, cellulose–glycerol interactions may have contributed to charge transport and conductivity along the non-conductive PDMS substrate. Furthermore, the crosslinked network formed in the polymer matrix ostensibly provides mechanical stability and enables the conductive properties to support potential bioelectronic applications. The attained SEM images results confirm that the porous, networked composite structure is successfully achieved and in which the cellulose and glycerol are incorporated successfully into the PDMS backbone, creating a modified surface with enhanced functional properties.

### 3.2 Electrochemical characterization

To optimize the composition of the fabricated nanocomposite electrodes, a series of samples were prepared with varying concentrations of α-cellulose additives, ranging from 15% to 33% by weight. This systematic variation aimed to identify the ideal proportion of cellulose that would maximize the functional performance of the electrodes, particularly in terms of their electrical properties. The specific compositions and corresponding performance metrics for each formulation are summarized in Table 1. Electrical characterization of these samples involved measurement of bulk resistance (obtained from EC-lab), electrical conductivity, and charge storage capacity (CSC). Bulk resistance was determined using electrical impedance spectroscopy (EIS), from which the conductivity of the materials was calculated. Cyclic voltammetry (CV) was performed to assess the charge storage behaviour of each sample, offering insights into their potential applicability in energy storage and bioelectronic interfaces. We estimated the conductivity (*σ*) from the following equation as performed in previous literature studies.^5^1
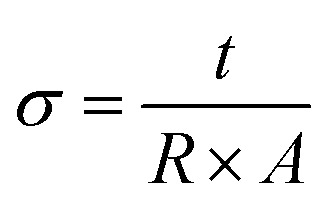
where *t* is the thickness of the sample, *R* is the bulk resistance from EIS and *A* is the cross-sectional area of the sample. The charge storage capacity (CSC) is estimated by integrating area below baseline of the cyclic voltammetry curve.This involved integration of the cathodal current density from the CV curve and dividing the resulting value by the sweep rate.^19,20^ The formula for the estimating the CSC values is as described in eqn (2) as estimated in the literature studies.^19,21^2
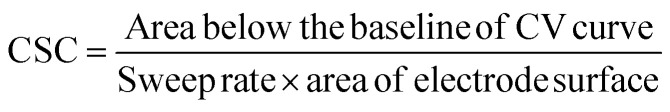


**Table 1:** Summary of electrochemical performance in comparison with literature

Sample 1	Cellulose (%)	PDMS	Glycerol (%)	Bulk resistance (KΩ)	Conductivity (S m^−1^)	Charge storage capacity (mC m^−2^)	References
1	15	77	8	3.07	4.23 × 10^−3^	—	This work
2	20	73	7	0.815	1.52 × 10^−2^	0.385	This work
3	23	70	7	0.732	1.72 × 10^−2^	0.589	This work
4	27	66	7	0.956	1.33 × 10^−2^	0.154	This work
5	28	66	7	0.975	1.30 × 10^−2^	0.484	This work
6	30	64	6	0.658	1.93 × 10^−2^	4.626	This work
7	33	61	6	0.774	1.64 × 10^−2^	4.531	This work
8	Mxene/PDMS			0.111	0.15	1.99	24
10	PDMS/MWCNT			—	3 × 10^−3^	—	22
11	PDMS/SNC/CNT			—	2.77	—	25
12	PANI–silicone-based			0.6	0.005 × 10^−2^	1.49	26
13	Silicone/titanium dioxide (TiO_2_)/PMMA			2.28	0.001	1.23	21

The electrical conductivity of the fabricated cellulose/PDMS composite electrodes was comprehensively analysed to test their potential as electrode flexible electronics. The electrochemical parameters, including conductivity, bulk resistance, electrochemical stability, and charge storage capacity (CSC), were measured for the uniformly thick 1 mm bioelectrode. These were ascertained by electrical impedance spectroscopy (EIS) and cyclic voltammetry (CV) results, after applying standard techniques to the electrode under test as described in the previous section. Table 1 enlists a summary of the electrochemical performance of the bioelectrode in comparison with the literature. The conductivity increases steadily from 4.23 × 10^−3^ S m^−1^ at 15% cellulose to a peak of 1.93 × 10^−2^ S m^−1^ at 30% cellulose. This trend confirms that higher cellulose content enhances the formation of interconnected hydrophilic domains, which, when combined with glycerol, create more favourable conditions for ion mobility within the otherwise non-conductive PDMS matrix. The most promising electrode of the series of the fabricated α-cellulose–PDMS–glycerol bioelectrodes exhibited a very low 0.658 kΩ bulk impedance and conductivity of 1.93 × 10^−2^ S m^−1^, indicating good ionic conductivity and minimal resistive losses within the electrode matrix. This was attained for 30% α-cellulose/64% PDMS/6% glycerol composition. Furthermore, this sample exhibited 4.6226 mC per m^2^ CSC, which indicates how efficiently the sample can store and deliver charge—a very important parameter for signal acquisition and electrical stimulation applications. Apart from this the CV voltammograms depicted stable oxidation/reduction curves without the presence of peaks which confirms the overall stability of the fabricated electrode. The conductivity attained from this study is comparable to that multiwalled carbon nanotube (MWNT)/PDMS composite from the literature 3 × 10^−3^ S m^−1^ (ref. 22) as enlisted in the comparison Table 1.

**Fig. 5:**
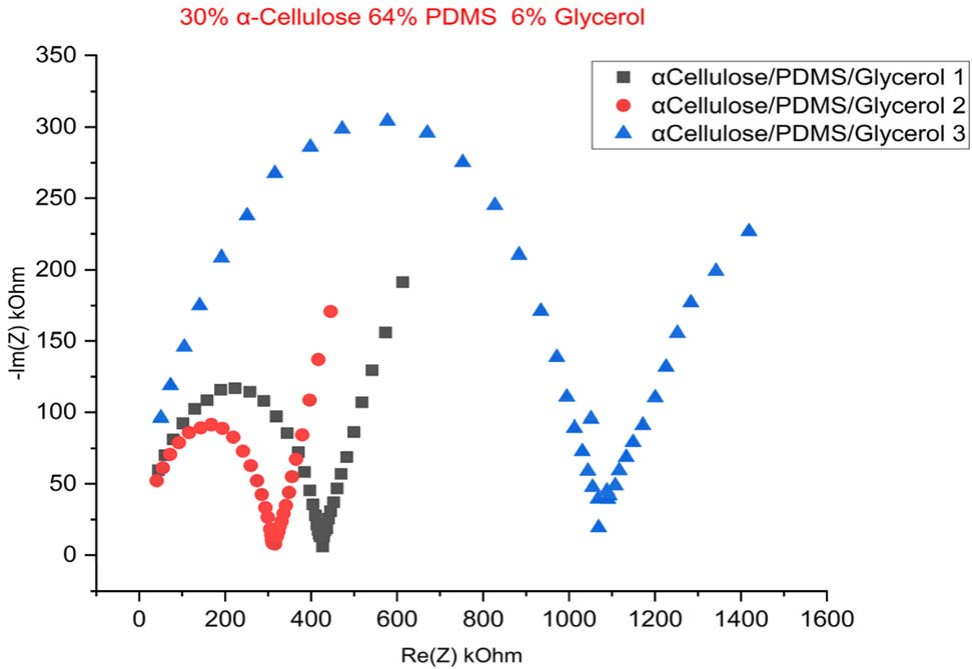
EIS data of the 30% cellulose 64% PDMS 6% glycerol triplicate samples.

**Fig. 6:**
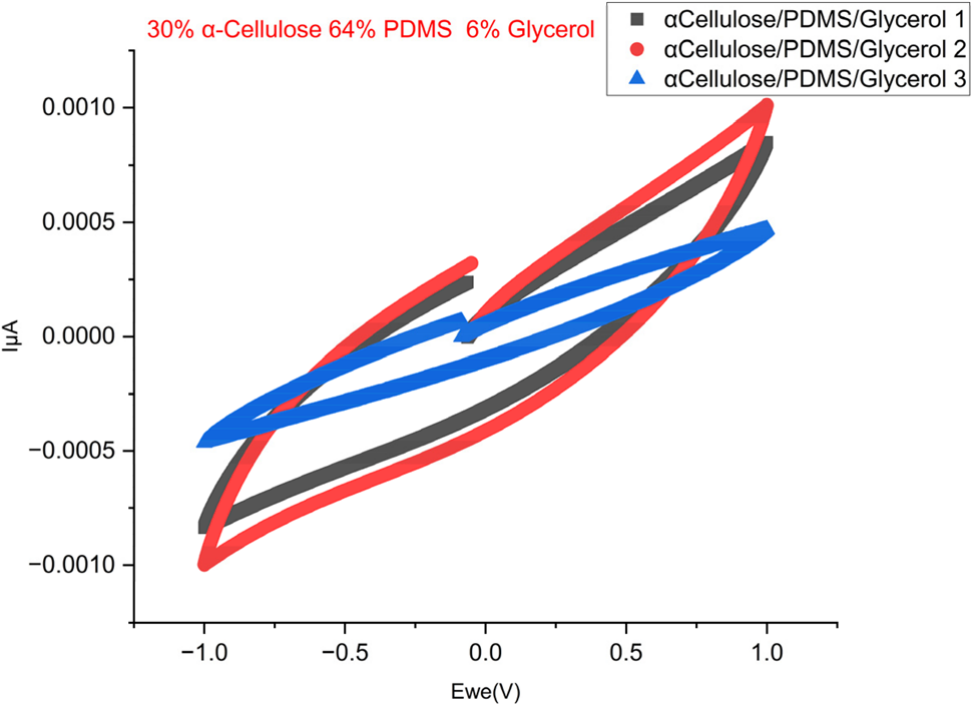
CV data of the 30% cellulose 64% PDMS 6% glycerol triplicate samples.

Fig. 5 shows Nyquist plot of the EIS test, which has a distinct semicircle at high frequencies, corresponding to the charge transfer resistance, followed by the linear Warburg regime at low frequencies, corresponding to ion diffusion within the porous electrode structure.^23^Fig. 6 is the cyclic voltammogram of the best sample (of a triplicate batch of the 30% α-cellulose/64% PDMS/6% glycerol), which exhibits a stable and symmetrical shape for multiple cycles, pointing to good capacitive behaviour and electrochemical stability. The incorporation of glycerol likely appears to act with cellulose by increasing water retention and polymer chain mobility, further improving ion transport. Compared to typical biocompatible soft materials used in bioelectronics, these conductivity values fall within an acceptable range for low-power signal transmission, particularly for epidermal or wearable electrodes. The achieved values, while not in the range of metallic conductors, are significant for ionic conduction, making the material suitable for applications such as ECG, EMG, or EEG signal acquisition where moderate conductivity and biocompatibility are prioritized. The results so far show that the proposed material has potential as a soft, high-performance bioelectrode for electrophysiological monitoring applications.

### 3.3 Mechanical characterization

To evaluate the elastic behaviour of the synthesized electrode materials, stress–strain analysis was conducted, and the collected data were utilized to construct stress–strain curves for every sample. The curves are important in identifying the mechanical behaviour of materials when subjected to applied stress, particularly their ability to deform elastically before yielding or failure. The most significant mechanical property of the stress–strain curve is Young's modulus, a measure of a material's flexibility and stiffness. It is determined as the slope of the linear (elastic) region of the stress–strain curve, in which stress is directly proportional to strain. The greater the slope, the stiffer the material; the smaller the slope, the more flexible the material. For the present investigation, rectangular pieces of the composite electrodes were well prepared according to standard sizes—50 mm length, 10 mm width, and 1 mm thickness. The samples were loaded in uniaxial tension under controlled conditions *via* a universal testing machine. Tensile load applied and the corresponding elongation of the material were noted to determine the stress–strain curve. In this study we chose the best performing electrode from the electrical characterization result to undergo mechanical testing. Here, we report the results of triplicate sample from the electrode composition of 33% cellulose/64% PDMS and 6% glycerol. Fig. 6 shows the plotted stress–strain curve of the electrode sample used to determine the Young's modulus through linear regression of the elastic region of the curve. This parameter is necessary in representing the mechanical strength and potential flexibility of the composite material, which is vital for its integration into wearable and stretchable electronic devices. A study by Chen *et al.* suggests that wearable electronics applications much possess a Young's Modulus lower that 25 kPa to embed skin like feature.^27^ The Young's modulus of skin and native tissues falls in the range of 2–25 kPa.

Table 2 provides summary of comparative results from current study and literature. The results of the mechanical characterization reveal that the best performing cellulose-based electrode (33% cellulose/64% PDMS/6% glycerol) has low Youngs Modulus in the range of 5–16 kPa which is much closer to that of skin tissues^28^ and conformable hydrogels^28^ from the literature. On overall, they have a great potential to be embedded in wearable sensing applications that require low Youngs modulus. According to Yu *et al.*, a low modulus of wearable devices provides better confirmability and comfort for health monitoring applications.^29^ For applications involving neural interfaces, soft materials are vividly employed for enhanced and stable connections with the nervous system while reducing potential side effects.^30^

**Table 2:** Comparative mechanical testing results from the literature

Composition	Youngs modulus	References
33% cellulose/64% PDMS/6% glycerol	10.3 ± 5.4 kPa	This work
PDMS/glycerol/BACD	9.8 ± 2.3 kPa	5
PAAm hydrogel	5.2 kPa	28
Microfiber-shaped neural probes	7.9 ± 3.1 kPa	30
PEDOT: PSS/PVA/PAA	12.7 kPa	31
Arm skin	7–22 kPa	32
Native brain tissue	2.7–3.1 kPa	33

**Fig. 7:**
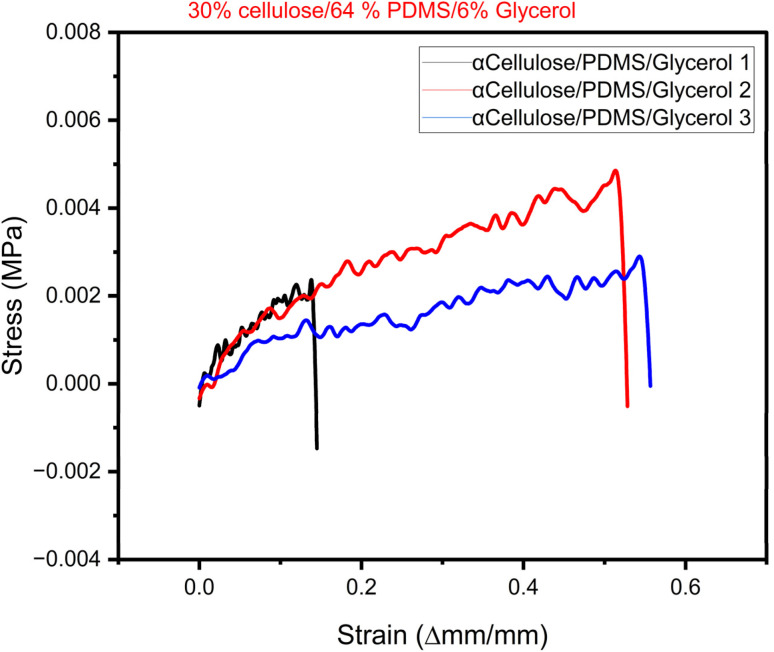
Stress–strain curve of the 33% cellulose/64% PDMS/6% glycerol triplicate sample.

The mechanical characterization results show that the best-performing cellulose-based electrode (30% α-cellulose/64% PDMS/6% glycerol) possesses a low Young's modulus of 5–16 kPa. Fig. 7 shows that the first sample of 30% α-cellulose/64% PDMS/6% glycerol composition tend to break faster in comparison to other samples. This may be due to subtle difference in the sample which may have been contributed by varying drying rate or component distribution. However, this can be prevented by stabilizing this in the future studies. The attained results are remarkably close to that of natural skin tissues and conformable hydrogels, as previously reported.^28^ Such a low modulus is particularly advantageous for application in wearable sensing technologies, where mechanical compatibility with human skin is critical. Materials that mimic the softness and flexibility of biological tissue are more likely to provide seamless integration, minimal pain, and improved user experience. The wearable application prospect of these cellulose-based electrodes is also indicated by a study by Yu *et al.*,^29^ which emphasized that low-modulus wearable devices are more conformable and comfortable and are therefore appropriate for continuous health monitoring. A soft interface provides a better interface with the skin, which can enhance the accuracy of physiological signal detection and reduce the likelihood of mechanical irritation with long-term use. Also, in neural interface use, soft, tissue-like material with mechanical properties has mainly been used to provide long-term, stable interaction with the nervous system. As noted in the literature,^30^ the use of compliant materials reduces the mechanical mismatch between implanted devices and surrounding neural tissue, minimizing inflammation, scarring, and other side effects. Collectively, these findings highlight the significant potential of the synthesized cellulose/PDMS electrodes for wearable health monitoring systems and bioelectronic interfaces. The reduced Young's modulus not only enhances user comfort but also contributes to functional performance and biocompatibility essential for future biomedical devices.

### 3.4 Recording ECG signals

**Fig. 8:**
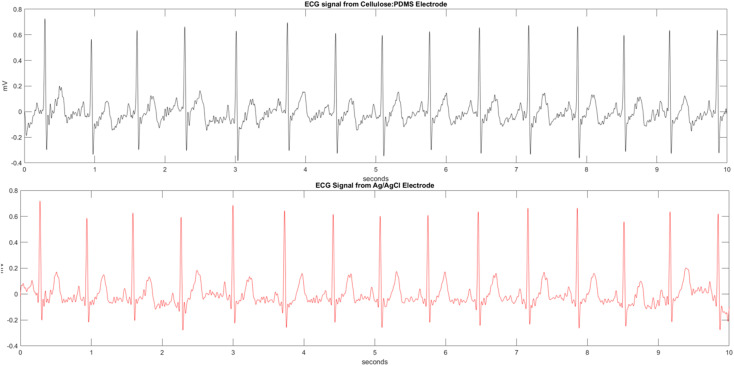
ECG signal recording from commercial electrode Ag/AgCl *vs.* fabricated electrode (30% α-cellulose, 64% PDMS glycerol, and 6% glycerol).

Finally, to determine the practical usefulness of the optimized cellulose-based composite electrode (30% α-cellulose, 64% PDMS, 6% glycerol), a comparison with the commercial standard Ag/AgCl electrode was carried out for recording electrophysiological signals. Specifically, a comparison of the performance of the composite electrode with that of recording ECG signals was carried out. For this purpose, the best-performing cellulose/PDMS/glycerol electrode composition (30% cellulose, 6% glycerol, and 64% PDMS by weight) was cast over a stainless-steel backing plate, mimicking that of commercially available Ag/AgCl electrodes. ECG signals were recorded using a PowerLab 26T data acquisition system in the standard Lead II configuration. The sampling rate was set at 1 kHz for high-resolution signal recording. The recorded data were compared with simultaneously recorded signals using the commercial Ag/AgCl electrodes. As can be seen from Fig. 8, both electrode ECG waveforms show the typical features of a normal ECG signal, including the P-wave, QRS complex, and T-wave, indicating stable signal detection and acquisition with the cellulose/PDMS-based electrode. From the comparative signal analysis, the fabricated cellulose-based electrode could capture high-quality ECG signals with minimal distortion or baseline drift. The estimated signal-to-noise ratio (SNR) of the cellulose/PDMS/glycerol electrode is 33.21 dB, which is only slightly lower than the 34.95 dB SNR obtained from the commercial Ag/AgCl electrode. These results affirm the potential of the fabricated cellulose/PDMS/glycerol electrodes as a sustainable and eco-friendly alternative to traditional metal-based electrodes for wearable and long-term electrophysiology monitoring applications.

The α-cellulose/PDMS/glycerol composite electrodes synthesized in this study exhibited good potential for electrophysiological applications, especially in the acquisition of ECG signals. One of the most important findings of this work is the electrochemical stability and electrical conductivity of the composite, as shown by cyclic voltammetry (CV) tests and EIS measurements. The presence of α-cellulose, which is a natural polymer rich in hydroxyl (–OH) groups, has significantly enhanced charge transfer in the material. This was also confirmed in the ATR-FTIR spectrum. With glycerol as a plasticizer, mechanical properties have been enhanced along with ion conduction paths, leading to decreased interfacial resistance. This resulted in high conductance and low bulk impedance of 0.0193 S m^−1^ for 30% α-cellulose/64% PDMS/6% glycerol composition as shown by EIS results. The electrode material has also demonstrated high mechanical flexibility, which is crucial for any wearable biosensor applications to ensure comfort. The soft and elastic properties of PDMS, coupled with α-cellulose material, produced an elastic electrode that is comparable with human skin (generally within the modulus range of 2–25 kPa (ref. 32)). This ensures a close match to the skin without inducing discomfort or causing rigidity as seen in metal-based electrodes. Altogether, the aforementioned properties enhanced electrochemical and mechanical properties of the electrode material to yield considerably improved ECG signal acquisition as seen by the signal recorded from the electrode composition of 30% α-cellulose, 6% glycerol, and 64% PDMS. In comparison to conventional metal electrodes, the composite provided a good quality signal with decreased motion artifacts. This improvement in signal recording can be also linked to the stable and flexible interface between the electrode and skin.

## 4. Conclusions

A novel α-cellulose/glycerol incorporated in PDMS substrate were successfully fabricated and their surface morphology, electrochemical, and mechanical characteristics were estimated. Morphological, electrochemical, and mechanical evaluations as a whole highlight the potential of these composites for next-generation wearable and bioelectronic devices. SEM observation revealed a porous and interconnected surface structure, which implies excellent integration between cellulose fibers and PDMS. Electrical impedance spectroscopy of cyclic voltammetry also validated the conductive nature of the composite, with the sample with the highest performance having a conductivity of 0.0193 S m^−1^ and a charge storage capability of 4.626 mC m^−2^. The composite electrodes were also mechanically compromised, with a Young's modulus in the range of 5–16 kPa, almost identical to soft biological tissues such as skin and hydrogels. This is a crucial property in giving comfort, flexibility, and long-term biocompatibility for wearables and neural interfaces. The porous electrode structure revealed from SEM images and low elastic modulus contributes to intrinsic compatibility with biological tissues, providing mechanical properties that closely mimic those of native tissue.^34^ Softness and stability of the electrodes render them appropriate for continuous health monitoring applications where conformability and comfort of the user are key considerations. To conclude, the work provides promising potential of glycerol-loaded cellulose/PDMS composites that offer an environmentally friendly, biocompatible, and excellent platform for fabrication of flexible bioelectrodes. Future study will aim to further optimize the composition of materials, explore long-term stability, and verify performance under real-world sensing conditions. In addition, the mechanical properties of the composite can be further enhanced to induce self-adhesiveness—an added feature essential for wearable sensors, as it helps minimize skin contact impedance.

## Author contributions

Amani Al-Othman: conceptualization, supervision, data curation, formal analysis, investigation, methodology, validation, visualization, and writing – editing and review. Meera Alex: data curation, formal analysis, investigation, methodology, validation, visualization, and writing – original and review. Mohammad Al-Sayah: formal analysis, investigation, methodology, validation, visualization, and writing – review and editing. Hasan Al-Nashash: conceptualization, resources, and writing – review and editing.

## Conflicts of interest

There are no conflicts to declare.

## Data Availability

The authors declare that the data supporting the findings of this study are available within the paper. Should any raw data files be needed in another format they are available from the corresponding author upon reasonable request.
